# Treating refractory obsessive–compulsive disorder with transcranial direct current stimulation: An open label study

**DOI:** 10.1002/brb3.1648

**Published:** 2020-05-14

**Authors:** Ghina Harika‐Germaneau, Damien Heit, Armand Chatard, Berangere Thirioux, Nicolas Langbour, Nemat Jaafari

**Affiliations:** ^1^ Unité de Recherché Clinique Intersectorielle en Psychiatrie à vocation régionale Centre Hospitalier Henri Laborit Poitiers France; ^2^ Université de Poitiers Poitiers France; ^3^ Laboratoire de Neurosciences Expérimentales et Cliniques INSERM U 1084 Poitiers France; ^4^ Laboratoire CeRCA CNRS 7295 Poitiers France

**Keywords:** obsessive–compulsive disorder, supplementary motor area, tDCS, treatment

## Abstract

**Background:**

Obsessive–compulsive disorder (OCD) is a complex disorder with 40%–60% of patients' refractory to treatment. Transcranial direct current stimulation (tDCS) has been shown to induce potent and long‐lasting effects on cortical excitability. The aim of the present clinical trial was to evaluate the therapeutic efficacy and tolerability of cathodal tDCS over the supplementary motor area (SMA) in treatment‐resistant OCD patients.

**Methods:**

Twenty‐one treatment‐resistant OCD outpatients received 10 sessions of tDCS. Each treatment session consisted of 2 mA stimuli for 30 min. The cathode was positioned over the bilateral SMA and the anode over the right supraorbital area. Patients were evaluated at baseline, end of treatment, one‐month follow‐up, and three‐month follow‐up. Response to treatment was defined as at least a decrease of 35% on the Yale–Brown Obsessive–Compulsive Scale (YBOCS) and a score of 2 or less on the Clinical Global Impressions‐Improvement (CGI‐I) between baseline and 1‐month follow‐up.

**Results:**

There was a significant decrease of YBOCS scores between baseline and one‐month assessment. At one month, five patients (24%) were considered as responders and 3 (15%) at 3 months. We also observed concomitant changes in depressive symptoms, and insight. The treatment was well tolerated. Short‐lasting side effects were reported as localized tingling sensation and skin redness.

**Conclusion:**

Our results suggest that the use of cathodal tDCS over the SMA and anodal tDCS over the right supraorbital area in OCD treatment‐refractory patients is safe and promising to improve obsessive and compulsive symptoms. Large randomized controlled trials are needed to confirm this positive result.

## Graphical abstract

Obsessive–compulsive disorder (OCD) is a complex disorder with 40%–60% of patients refractory to treatment. Transcranial direct current stimulation (tDCS) will offer a new insight in OCD treatment. The aim of the present open clinical trial was to evaluate the therapeutic efficacy and tolerability of cathodal tDCS over the supplementary motor area in treatment‐resistant OCD patients.

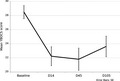

## 1 INTRODUCTION

Obsessive–compulsive disorder (OCD) is a multifaceted, debilitating neuropsychiatric disorder associated with serious cognitive and behavioral impairments that affects social function and quality of life (Coluccia et al., 2016). The most frequent clinical symptoms of OCD are contamination obsessions and washing/cleaning compulsions, both of which are characterized by recurring intrusive thoughts. The prevalence of OCD is around 2%–3%; onset occurs either during infancy or young adulthood (Ruscio, Stein, Chiu, & Kessler, 2010). The usual treatment of OCD is serotonin reuptake inhibitors (SRIs) coupled with cognitive behavioral therapy (CBT; Hirschtritt, Bloch, & Mathews, 2017). Despite improvements in pharmacological treatment and psychotherapy, 40%–60% of patients endorse residual and impairing symptoms (Skapinakis et al., 2016). Therefore, it is important to develop alternatives to conventional therapies. Neuromodulation techniques offer promising avenues for treatment.

Neuromodulation techniques may be invasive, such as deep brain stimulation, or non‐invasive, like repetitive transcranial magnetic stimulation (rTMS) and transcranial direct current stimulation (tDCS). These techniques aim to modify activity and connectivity within brain networks. Over the last decade, rTMS has received a great deal of attention (Rehn, Eslick, & Brakoulias, 2018). However, further research is needed to gauge the efficacy and safety of modern tDCS in treating OCD. The present open label study aims to contribute to the extant literature by investigating the effectiveness of tDCS over the supplementary motor area (SMA) in treatment‐resistant OCD patients.

tDCS is a simple, low‐cost technique that has robust safety and tolerance with emerging evidence for its efficacy under certain psychiatric conditions (Kekic, Boysen, Campbell, & Schmidt, 2016). tDCS consists of applying a direct electric current across two flat, large electrodes placed on the scalp. Electrodes are classified as “anodal” or “cathodal,” given the polarity of the active electrode, which is placed over a targeted cortical region. When applied over the motor cortex, tDCS can either increase (anodal stimulation) or decrease (cathodal stimulation) motor cortex excitability (Nitsche & Paulus, 2000) and regional cerebral blood flow (Zheng, Alsop, & Schlaug, 2011). This electric current can enter the skull and reach the cerebral cortex, thereby modifying the neuronal membrane's resting potential and modulating the neuronal firing rates (Chhatbar et al., 2018). tDCS increases cortical excitability without inducing an action potential (Nitsche & Paulus, 2001). The suggested capacity of tDCS to enlarge recovery of brain function, by promoting learning and facilitating plasticity, has motivated the development of several clinical trials regarding psychiatric disorders (Kuo, Chen, & Nitsche, 2017). Overall, the results of these initial studies show promising outcomes for depression, addiction, cravings, and auditory or verbal hallucinations in schizophrenia.

Concerning OCD, the use of tDCS is relatively scarce. In recent years, a limited number of controlled and open label studies as well as case series have been published on the safety and efficacy of tDCS for treatment of OCD (Brunelin et al., 2018; Rachid, 2018). To date, there are only three published randomized trials on this topic (Bation, Mondino, Camus, Saoud, & Brunelin, 2019; D’Urso et al., 2016; Gowda et al., 2019). Based on neuroimaging data, six different electrode montages were tested, with interesting effects on reducing obsessive–compulsive symptoms (for review, see Brunelin et al., 2018). Despite encouraging results in OCD, to date there are no optimal target locations or stimulation parameters; further data are urgently needed.

The dominant neurobiological model of OCD has implicated dysfunctional cortico–striato–thalamo–cortical (CSTC) circuits, including the medial prefrontal cortex (i.e., SMA), anterior cingulate cortex, orbitofrontal cortex (OFC), and the basal ganglia in the etiology of clinical symptoms and cognitive deficits (Nakao, Okada, & Kanba, 2014; Piras et al., 2015). Among these, the OFC (Ahmari & Dougherty, 2015; Niu et al., 2017) and the SMA (Grützmann et al., 2016; de Wit et al., 2012) seem to be particularly relevant, as demonstrated by several neuropsychological and neuroimaging investigations.

In line with the prevalent neurobiological model of OCD, we reasoned that targeting the SMA with cathodal tDCS, coupled with anodal tDCS over the right supraorbital area, may reduce obsessive and compulsive symptoms by modulating neuronal activity within the orbitofronto‐striato‐pallido‐thalamic loop. A recent open label study with a similar electrode montage found significant improvement in obsessive and compulsive symptoms one week after 20 tDCS sessions in a sample of 20 treatment‐resistant OCD patients (Kumar, Kumar, & Verma, 2019). The present study aimed to replicate and extend this promising finding by testing whether tDCS effects persist over a longer time.

In the present open label study, we expected tDCS protocol with cathodal over the SMA and anodal stimulation over the right supraorbital area to be efficient in reducing OCD symptoms in treatment‐refractory patients with effects lasting 1–3 months after the treatment.

## 2 MATERIAL AND METHODS

### 2.1 Study design

We performed a 2‐week open label study of tDCS. The trial was conducted in the Poitiers Henri Laborit psychiatric hospital in France. Ethical clearance was obtained from the Institutional Review Board of CPP Ouest III Poitiers (Approval number: 15.12.55), and trial registration was done with the Clinical Trial Registry before the study began (ClinicalTrials.gov Identifier: NCT03284671). All patients provided written informed consent after a full description of the study and potential tDCS adverse effects.

### 2.2 tDCS procedure

Each patient received a total of 10 tDCS sessions, which were delivered once a day, 5 days a week, for 2 weeks (from Monday to Friday). Stimulation sessions were done using an HDCKit NEWRONIKA S.r.l (Via Dante, 4‐20121 MILANO‐ITALY, CE0068). The stimulator was connected to two rubber electrodes (7 × 5 cm, 35 cm^2^) placed inside a sponge, which, in turn, was soaked on each side in a saline solution (0.9% NaCl) and fixed over the sites of interest with a tubular net bandage.

A typical session of tDCS consisted of delivering a direct current of 2 mA for 30 min. Electrodes were positioned on the scalp following the international 10–20 electrodes placement system. The cathode was placed on the sagittal midline at 15% of the distance between inion and nasion anterior to Cz, using the International 10–20 EEG System to target the bilateral SMA (Mantovani, Simpson, Fallon, Rossi, & Lisanby, 2010). The anode was placed over the right orbitofrontal area above FP2, according to the 10–20 international systems for EEG. During the tDCS session, patients were instructed to relax and stay awake with open eyes.

### 2.3 Participants

Patients were recruited between February 2016 and May 2017. Twenty‐one outpatients aged between 18 and 70 years, with DSM‐IV‐TR OCD, diagnosed using the Mini‐International Neuropsychiatric Interview (MINI; Sheehan et al., 1998) were enrolled in the study. To be eligible, patients were required to have a total YBOCS score of 20 or more, total duration of disease of at least 2 years, and should have received at least two 12‐week treatments with SRIs and CBT but were not responding (treatment‐refractory). The current medication regimen was maintained throughout the treatment and follow‐up visits. Benzodiazepines (lorazepam, clorazepate, oxazepam, verapamil, or alprazolam) were also maintained at the same dose throughout the study.

Exclusion criteria were as follows: a diagnosis of schizophrenia, current major depressive disorder (Montgomery–Asberg Depression Rating Scale (MADRS) > 21), other psychotic disorders, bipolar I disorder, substance and alcohol dependence within the last 6 months; suicidal (score of 3 or more in MADRS, moderate or severe stage in MINI); severe or unstable medical conditions; or history of epilepsy.

### 2.4 Assessment

Trained psychiatrists completed the clinical assessments. All assessments included the YBOCS, Clinical Global Impressions‐Severity (CGI‐S), Clinical Global Impressions‐Improvement (CGI‐I), MADRS, Brown Assessment of Belief Scale (BABS), Hospital Anxiety and Depression scale (HAD), and the Short Form health survey (SF‐36). Patients were assessed at baseline, post‐tDCS treatment (14 days after baseline), after 1‐month follow‐up (45 days after baseline), and 3‐month follow‐up (105 days after baseline). Both patients and psychiatrists administrating evaluation were aware of treatment statues.

The safety of tDCS was assessed after each session using a structural interview (Brunoni et al., 2011).

### 2.5 Outcome measures

The primary outcome measure was the total YBOCS score. Responder status was defined as at least a decrease of 35% on the YBOCS and a score of 2 or less on the CGI‐I (much or very much improved) between baseline and 1‐month follow‐up and remission is indicated as a score of ≤12 on the YBOCS plus CGI‐S rating of 1 (“normal, not at all ill”) or 2 (“borderline mentally ill”; Mataix‐Cols et al., 2016).

The secondary outcome measures were the change in severity rating score on the MADRS, BABS, CGI‐S, CGI‐I, HAD, and SF‐36 at 1‐month follow‐up, and the change in YBOCS, MADRS, BABS, CGI‐S, CGI‐I, HAD, and SF‐36 at 3‐month follow‐up.

### 2.6 Statistical analysis

All statistical analyses were performed using JASP (https://jasp‐stats.org). Our primary outcome was the score obtained by YBOCS at the 4 assessment times (before tDCS, after 10 sessions of tDCS, 1 and 3 months later). Following the study protocol, only patients with a baseline assessment and at least one post‐tDCS score (during the 3‐month follow‐up period) were considered in the statistical analyses. The analyses were conducted in a last‐observation carried‐forward manner through the endpoint time. The significance level was set at *p* < .05. Analyses of variance (ANOVAs) for repeated measures were conducted with the within‐subject factor of time. In case Mauchly's sphericity test was significant, ANOVA results were adjusted for sphericity using the Greenhouse‐Geisser correction. The significance level was set at *p* < .05. We used Bonferroni adjustments of alpha levels to account for multiple comparisons. To examine whether effects of tDCS on depressive symptoms were caused by concomitant changes in obsessive and compulsive symptoms, we also ran two regression analyses to examine whether effects of tDCS on depressive symptoms were caused by concomitant changes in obsessive and compulsive symptoms.

## 3 RESULTS

### 3.1 Participants

All patients (13 males and 8 females, mean age 42.7 ± 13, age of onset 14.9 ± 8.4, duration of illness 27.3 ± 11.7) completed the 10 stimulation sessions, the post‐tDCS assessment (14 days after baseline), and 1‐month follow‐up assessment (day 45). Only 15 patients completed the last assessment visit (day 105). At baseline, seven (33%) patients were drug‐free, and 13 (62%) patients had stable pharmacological treatment. Their treatments involved SSRIs (*n* = 9), clomipramine (*n* = 1), other antidepressants (*n* = 1), association with SSRI and clomipramine (*n* = 2), antipsychotics (*n* = 6), anti‐histamine agents (*n* = 1), and benzodiazepines (*n* = 9). The patients’ treatments were maintained throughout the study. Seven (33%) of the 21 patients had current augmentation treatment with a combination of an antidepressant and either another antidepressant or an antipsychotic. All patients had tried CBT in the past.

Overall, tDCS treatment was well tolerated. There were no major clinical or cognitive side effects during the 10 tDCS sessions. The most common side effect was a mild tingling sensation (62.4%), followed by skin redness (45.7%), burning sensation (19%), sleepiness (15.2%), itching (30.4%) and, less frequently, headache (11.4%), scalp pain (4.3%), trouble concentrating (2.9%), neck pain (1.9%), and acute mood change (0.5%). All side effects were mild, short‐lived, well tolerated, and spontaneously resolved. No severe adverse events were reported during the trial.

### 3.2 Responder status

Patients were classified as responders if they showed at least a decrease of 35% on the YBOCS and a score of 2 or less on the CGI‐I (much or very much improved). After the 10 tDCS sessions, 6 (28.5%) patients were responders (4 were drug‐free). At the 1‐month follow‐up (day 45), 5 (24%) patients were responders (1 was drug‐free). At the 3‐month follow‐up (day 105), 3 patients (15%) were responders (1 were drug‐free). Remission is indicated as a score of ≤12 on the YBOCS plus CGI‐S rating of 1 (“normal, not at all ill”) or 2 (“borderline mentally ill”; Mataix‐Cols et al., 2016). At the 1‐month follow‐up, 2 patients are considered as remitted and 1 patient at 3‐month follow‐up.

### 3.3 Primary outcome

A significant effect was observed on obsessive and compulsive symptoms, as assessed by the total YBOCS scores variation (Table 1).

**Table 1:** Repeated measures ANOVA results

Scales	Baseline *n* = 21	D14 *n* = 21	D45 *n* = 21	D105 *n* = 21	RM Stats
YBOCS	28.8 (4.3)	22.2 (6.2)	21.8 (7.2)	23.6 (6.7)	*F*(3, 60) = 12.335; *p* < .001
Obsessions	14.4 (3.0)	11.9 (4.4)	11.0 (3.8)	11.8 (3.7)	*F*(3, 60) = 10.923; *p* < .001
Compulsions	14.1 (2.7)	11.4 (3.1)	10.8 (4.0)	11.9 (4.2)	*F*(3, 60) = 12.335; *p* < .001
CGI‐S	5.0 (0.6)	4.8 (0.7)	4.6 (1.0)	4.8 (0.8)	*F*(1.9, 38.0) = 2.162; *p* = .131
CGI‐I		2.9 (0.7)	3.2 (0.8)	3.5 (0.7)	*F*(2, 38) = 6.257; *p* = .004
MADRS	14.3 (5.0)	9.5 (6.8)	9.9 (6.9)	11.2 (6.3)	*F*(3, 60) = 5.008; *p* = .004
BABS	3.3 (3.3)	2.8 (3.5)	1.7 (2.5)	1.5 (2.5)	*F*(3, 60) = 5.785; *p* = .002
HAD‐A	12.7 (4.1)	11.8 (4.7)	11.6 (4.0)	12.6 (4.3)	*F*(3, 60) = 0.992; *p* = .403
HAD‐D	11.0 (3.8)	9 (4.5)	9.2 (4.0)	9.7 (3.7)	*F*(3, 60) = 3.372; *p* = .024
SF36					
Physical Functioning	75.7 (23.5)	73.6 (25.7)	77.4 (22.2)	77.4 (21.1)	*F*(3, 60) = 0.649; *p* = .587
Role limitation due to physical heath	35.7 (45.8)	40.5 (44.3)	44.0 (43.9)	40.5 (42.9)	*F*(1.9, 39.3) = 0.619; *p* = .541
Bodily pain	69.6 (30.7)	71.8 (26.0)	69.6 (27.0)	68.4 (25.2)	*F*(3, 60) = 0.234; *p* = .872
General health	49.0 (21.9)	48.6 (21.3)	48.4 (21.9)	46.8 (22.0)	*F*(3, 60) = 0.314; *p* = .816
Vitality	27.6 (16.6)	30.7 (15.7)	35.2 (17.8)	33.3 (16.5)	*F*(1.9, 39.6) = 3.067; *p* = .058
Social Functioning	32.5 (20.0)	38.1 (20.9)	38.1 (20.5)	35.0 (20.1)	*F*(1.8, 34.4) = 0.956; *p* = .387
Role limitation due to emotional problems	13.3 (27.4)	21.7 (34.7)	23.3 (34.4)	21.7 (34.7)	*F*(1.8, 34.5) = 0.777; *p* = .456
Mental health	50.7 (11.0)	55.4 (10.0)	54.1 (11.0)	53.7 (10.6)	*F*(3, 60) = 2.240; *p* = .093

Note

Data are presented as mean (*SD*).

Abbreviations: BABS, Brown Assessment of Beliefs Scale; BAS, Brief Anxiety Scale; CGI‐I, Clinical Global Impressions‐Improvement; CGI‐S, Clinical Global Impressions‐Severity; HAD‐A, Hospital Anxiety and Depression scale Anxiety; HAD‐D, Hospital Anxiety and Depression scale Depression; MADRS, Montgomery–Asberg depression rating scale; RM stats, Analyses of variance for repeated measures; YBOCS, Yale–Brown Obsessive–Compulsive Disorder Scale.

**Figure 1:**
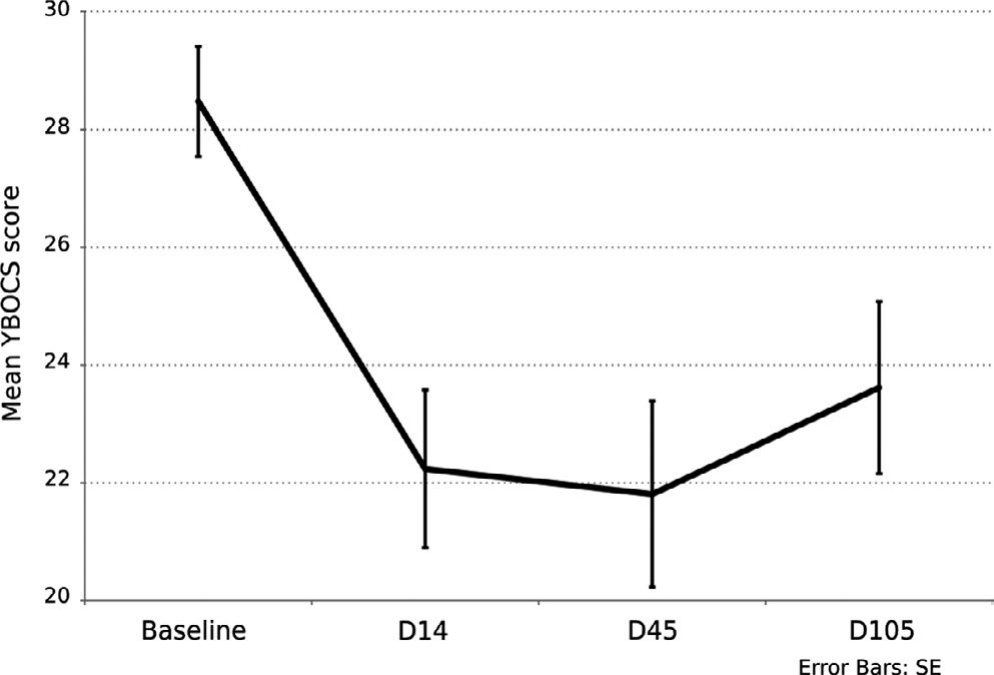
Evolution of Yale–Brown Obsessive–Compulsive Scale scores at baseline, after 10 tDCS sessions, and at 1‐ and 3‐month follow‐up. Results are given as mean ± *SE*

The beneficial effect was observed immediately after the 10 sessions of tDCS and lasted during the 3 months of the follow‐up period (baseline vs. D14 *t* = 6.684, *p*_bonf_ < .001; baseline vs. D45 *t* = 4.145, *p*_bonf_ = .003; and baseline vs. D105 *t* = 4.002, *p*_bonf_ = .004; Figure 1). The YBOCS total score of mean reduction was 26.4% (*SD* = 15.8). The beneficial effect was observed on both obsession (27%, *SD* = 3.6) and compulsion (26%, *SD* = 4.0) subscores of the YBOCS (Table 1).

### 3.4 Secondary outcomes

We observed a significant effect of tDCS on depressive symptom intensity, measured by MADRS scores and HAD‐D scores (*p* = .05 and *p* = .025). A significant effect was also observed on patient insight level modification as measured by BABS scores (*p* = .002) and on clinical global impressions‐improvement (*p* = .05). No significant effect was observed on the HAD anxiety scale (*p* = .403) and quality of life, evaluated by the SF‐36 instrument (Table 1).

### 3.5 Regression analysis

In the first regression analysis, the mean difference in MADRS scores between before and after the treatment (day 45) was predicted by the mean difference in YBOCS scores (baseline day 45). In this model, the effect of the intercept was not significant *B* = 0.57, *SE* = 1.81, *t*(19) = 0.31, *p* = .757, after control for the effect of YBOCS scores, *B* = 0.57, *SE* = 18, *t*(19) = 3.12, *p* = .006. In the second analysis, the mean difference in YBOCS scores between before and after the treatment (day 45) was predicted from the mean difference in MADRS scores (baseline day 45). In this model, the effect of the intercept was significant, *B* = 4.06, *SE* = 1.57, *t*(19) = 2.57, *p* = .019 after control for MADRS scores, *B* = 0.57, *SE* = 0.18, *t*(19) = 3.12, *p* = .006. Results of these analyses showed an improvement in obsessive and compulsive symptoms following tDCS treatment accounted for the (mediated) observed change in MADRS scores, rather than the other way around.

## 4 DISCUSSION

The key finding of this open label study was that 10 sessions of cathodal tDCS over the bilateral SMA significantly reduced OCD symptoms in treatment‐resistant patients. In this trial, 5 patients (24%) could be qualified as responders at the endpoint. Thus, cathodal stimulation over the bilateral SMA and anodal stimulation over the right supraorbital area with bipolar tDCS appear to be a safe, and interesting approach to improve OCD symptoms.

The SMA is an important cortical region implicated in OCD and is thought to mediate error monitoring and response inhibition along with other brain regions, such as the cingulum (Ridderinkhof, Ullsperger, Crone, & Nieuwenhuis, 2004). Neuroimaging studies suggest that the SMA is hyperactive in OCD patients, and this hyperactivity might relate to the deficient inhibitory control of behavior (Norman et al., 2018; de Wit et al., 2012). Therefore, the SMA can be a relevant brain stimulation target to modulate the subcortical regions and influencing OCD symptoms, especially in compulsive behaviors. Low‐frequency (1 Hz) rTMS studies (Mantovani et al., 2006, 2010) showed that inhibition of the SMA has a specific effect in reducing OCD symptoms. Inhibition of the SMA might cause suppression of the hyperexcitable right hemisphere and thereby improve dysfunctional symptoms in patients with OCD. Also, Senço et al., 2015) performed computational models to simulate electrode montages that target OCD brain regions. That study found the tDCS montage with the cathode over the pre‐SMA and extracephalic anode seems to activate most of the areas related to OCD.

Moreover, recent tDCS studies have reported an improvement in obsessive–compulsive symptoms after SMA cathodal stimulation with the anode placed in an extracephalic position over the lateral surface of the patient's deltoid (D'Urso et al., 2016; Silva, Brunoni, Miguel, & Shavitt, 2016) or over the right occipital area (Kumar et al., 2019). Kumar et al., in a very recent open label study, described an encouraging clinical effect with the same electrode montage. In that trial, 20 treatment‐resistant OCD patients received 20 tDCS sessions (2 sessions per day for 10 days) with the cathode over the SMA and the anode at the right occipital area. Kumar and colleagues described a tDCS acute positive effect with a significant decrease in total YBOCS scores between baseline and one week, following the last sessions. In this trial, the response was defined as at least a 35% reduction in YBOCS score. The author observed that three patients could be qualified as responders at the end point. In our trial, we use almost the same parameter (current dosage, duration of the stimulation, and electrode montage) except for the number of tDCS sessions and the duration of the follow‐up period. YBOCS reduction was almost the same in our trial after 10 sessions, and this encouraging effect was maintained at 1‐ and 3‐month follow‐ups.

Interestingly, this clinical effect may not be polarity‐dependent. Gowda et al. (2019) recently demonstrated that anodal stimulation over the SMA and cathodal stimulation over the right supraorbital area is effective in treating SRI‐resistant OCD patients. As such, the effects of tDCS may be obvious at both the anode and cathode as well as between the electrodes, thus modifying excitability over a much larger area of the cortex. Also, recent in vivo research offers direct evidence that tDCS can generate electric fields deep inside the brain (Chhatbar et al., 2018). In our study, the anode electrode over the right supraorbital area was used as an “inert” reference electrode. However, it is also possible to assign the clinical effect of tDCS to the anodal impact on the right supraorbital area. Therefore, it would be interesting in future trials to use neuroimaging and/or neurophysiologic techniques in addition to clinical assessments for a better understanding of tDCS’ neurobiological action.

In this study, we included 7 drug‐free patients. All seven had tried at least 3 SRI treatments in the past, antipsychotic association, and CBT without satisfactory clinical response. All patients expressed interest in a novel treatment option. After 10 tDCS sessions, 3 of them were responders, and 1 maintained this effect at 1‐month and 3‐month follow‐up. It could be suggested that the association of SRI with tDCS is superior to SRI or tDCS alone, and that SRI treatment induces a long‐lasting simulative effect of stimulation. This association was previously described in major depressive disorder (Brunoni et al., 2013).

Improvements in depression symptoms were also observed in our study. All participants showed signs of mild depression, with scores between 8 and 18 on the MADRS and no incidence of major depressive disorder. Improvements in OCD symptoms could possibly be secondary to a non‐specific antidepressant effect of tDCS. Despite this improvement, we found that changes in the YBOCS score did not correlate with changes in depression. Moreover, the fact that changes in YBOCS were independent of the baseline level of depression strengthens the hypothesis regarding a specific tDCS effect on OCD with a secondary improvement in depression. We also observed a significant improvement in patient insight after the tDCS sessions. This improvement was not correlated with a decrease in YBOCS scores.

OCD is associated with impaired quality of life and function (Coluccia et al., 2016). Thus, it is important to test whether tDCS may also lead to improvements in these essential outcomes. In the present trial, there was no significant improvement in the SF‐36’s dimensions after tDCS treatment. This result is at odds with other clinical studies conducted in OCD patients (Hollander, Stein, Fineberg, Marteau, & Legault, 2010; Velloso et al., 2018). Future studies are needed to determine when and why tDCS treatment is effective in improving perceived quality of life.

Two major limitations of this study could be addressed in future research. The first is the sample size. Even though our sample size is comparable to the sample used in other open label studies, including a larger sample of OCD patients will be an important asset in future studies. The second limitation concerns the absence of an active control condition. As the interpretation of the results is somewhat murky without a proper control condition, it should be essential to include a sham condition in future studies. Relatedly, our team is currently in the process of collecting the data of a large multisite double‐blind randomized clinical trial with the same tDCS parameters in order to confirm the efficacy and the safety of this cathodal stimulation over the SMA in treatment‐refractory OCD patients.

## 5 CONCLUSION

The current open label pilot study provides some evidence for the efficacy and safety of cathodal stimulation over the SMA and anodal stimulation over the right supraorbital area in treatment‐resistant OCD patients. Building on this recent effort, randomized clinical trials are needed to shed more light on the clinical effectiveness of this tDCS protocol.

## CONFLICTS OF INTEREST

The authors declare no conflicts of interest related to this study.

## AUTHOR CONTRIBUTIONS

DH, NL, GHG, and NJ conceived and supervised the study, GHG and DH recruited patients and gathered patient data, GHG, AC, BT, and NL retrieved the data, GHG wrote the manuscript, AC and BT correct the manuscript, GHG and NL revised the manuscript, all authors read and approved the final manuscript.

## Data Availability

The data that support the findings of this study are available from the corresponding author upon reasonable request.
